# DeepAEG: a model for predicting cancer drug response based on data enhancement and edge-collaborative update strategies

**DOI:** 10.1186/s12859-024-05723-8

**Published:** 2024-03-09

**Authors:** Chuanqi Lao, Pengfei Zheng, Hongyang Chen, Qiao Liu, Feng An, Zhao Li

**Affiliations:** 1 Research Center for Graph Computing, Zhejiang Lab, Yuhang, Hangzhou, 311121 Zhejiang China; 2 Department of Statistics, Stanford University, Stanford, Palo Alto, CA 94305 USA

**Keywords:** IC50, Graph convolutional network, Transformer, Drug response prediction, Data augmentation

## Abstract

**Motivation:**

The prediction of cancer drug response is a challenging subject in modern personalized cancer therapy due to the uncertainty of drug efficacy and the heterogeneity of patients. It has been shown that the characteristics of the drug itself and the genomic characteristics of the patient can greatly influence the results of cancer drug response. Therefore, accurate, efficient, and comprehensive methods for drug feature extraction and genomics integration are crucial to improve the prediction accuracy.

**Results:**

Accurate prediction of cancer drug response is vital for guiding the design of anticancer drugs. In this study, we propose an end-to-end deep learning model named DeepAEG which is based on a complete-graph update mode to predict IC50. Specifically, we integrate an edge update mechanism on the basis of a hybrid graph convolutional network to comprehensively learn the potential high-dimensional representation of topological structures in drugs, including atomic characteristics and chemical bond information. Additionally, we present a novel approach for enhancing simplified molecular input line entry specification data by employing sequence recombination to eliminate the defect of single sequence representation of drug molecules. Our extensive experiments show that DeepAEG outperforms other existing methods across multiple evaluation parameters in multiple test sets. Furthermore, we identify several potential anticancer agents, including bortezomib, which has proven to be an effective clinical treatment option. Our results highlight the potential value of DeepAEG in guiding the design of specific cancer treatment regimens.

## Introduction

Cancer continues to be one of the leading causes of death worldwide with its incidence showing an upward trend in recent years [1]. Developing new therapeutic drugs with a selective antitumor effect is both scientifically significant and clinically valuable. Due to the strong heterogeneity of cancer, similar anticancer drugs can induce different reactions in patients with the same type of cancer [2]. That highlights the significance of individualized cancer treatment, that is, based on patients’ genotype information and physiological characteristics, a precise drug regimen is recommended for patients to improve therapeutic effect and reduce drug side impacts [3, 4].

Particularly, cancer cell line(CCL) genomics plays an important role in personalized cancer drug design research. Cancer cell lines are a series of cell cultures isolated from patients and cultured in vitro [5], which are homologous to the primary tumor and are ideal models for studying tumor biology, pathogenesis, and drug sensitivity [6]. At the same time, the development of high-throughput sequencing technology [7] promotes the development and accumulation of cancer cell line database [8, 9]. Commonly data used in academic research databases such as Cancer Cell Line Encyclopedia (CCLE) [9] and Genomics of Drug Sensitivity in Cancer (GDSC) [8] have included a large amount of cell line genetic data, which provide researchers with a variety of omics data including genome, transcriptome, methylomic data, and quantitative indexes of drug response across cancer cell lines. Semi-maximum inhibitory concentration (IC50) is a widely used index. Analyzing the intrinsic characteristics of cancer-related genes and their interactions with anticancer drugs can reveal potential characteristics of anticancer molecules, thus simplifying the early screening of anticancer drugs, and improving the discovery efficiency of specific anticancer drugs.

Nowadays, numerous computational models have been devised for predicting cancer drug response by integrating omics characteristics with molecular descriptors and quantitative indicators [10]. The introduction of energy association in similarity-based data-driven models has proven beneficial for optimizing graph prediction tasks [11]. For instance, network-driven methods capture potential representations by constructing similarity networks [12, 13]. The dual-layer cell line-drug network model simultaneously constructs a cell and drug bisimilarity network, and then predicts drug response through a weighted model [12]. MOFGCN constructs heterogeneous networks by combining cell line similarity, drug similarity, and known cell line-drug association to learn potential cell lines and drug characteristics [13]. However, the effectiveness of these methods can be limited by their restricted generalization and computational efficiency. These factors can potentially affect their effectiveness and reliability in practical applications.

Methods of machine learning for cancer-drug prediction include but are not limited to logistic regression [14], support vector machines [15], multilayer neural networks [16] and random forests [17]. Besides, deep learning-based methodologies employ complex deep neural network architectures to extract intricate information from multi-source data. CDRscan adopts a two-step convolution architecture, which processes the mutation data of cell lines of drug molecular fingerprinting machine respectively and then performs virtual docking to complete the modeling [16]. The tCNNs uses dual convolutional neural networks to learn potential representations of drug and genomic mutation data respectively [18]. MOLI integrates multiple omics features through specific coding subnetworks for drug response prediction [19, 20]. Furthermore, DeepTTA utilizes transformers to facilitate drug representation learning and multilayer neural networks for transcriptomic data prediction, yielding superior performance [21].

The above methods have progressively evolved to incorporate a wider range of feature extraction techniques to address the increasing complexity of multi-omics integration. However, as data representation is constrained by certain limitations, some potential information might be inevitably lost during incomplete feature learning. For example, molecular fingerprints and SMILES are encoded based on molecular structure, so some molecular details such as stereoisomerism, charge distribution, and solvent effects are lost [22]. Recently, graph-based approaches have emerged as a promising solution, capitalizing on the natural ability of drugs to be represented as graphs and utilizing the power of graph convolutional networks (GCNs). DeepCDR use uniform graph convolutional network (UGCN) architecture to unify the characterization of drugs and integrate multiple omics features to achieve excellent prediction results [5]. GADRP constructs a sparse drug cell line pair (DCP) network containing similar information of drug, cell line, and DCP based on graph convolutional networks (GCNs) and autoencoder (AEs) to further improve the IC50 prediction performance [23]. Despite recent advancements, the incorporation and integration of molecular chemical bond information into the feature transfer process have been impeded by the intricate nature of molecular edge characterization and the constraints associated with updating methods in graph neural networks. Given the significance of molecular chemical bond information as a crucial component of molecular data, it is meaningful and necessary to optimize and improve the edge updating fusion algorithm.

We summarize the limitations of previous studies as follows:

- Exisiting works ignore the chemical bond information in a drug molecule, which is essential to distinguish the interaction between two chemical atoms. This information also has the potential to influence the outcome of drug-cancer cell line interactions directly.
- Previous works either applied string-based methods, such as SMILES or graph-based methods to represent drug molecules [24]. However, both these two methods can provide complementary information for drug discovery. Fully utilizing both information can help learning a better potential representation of drugs.
- Most previous works only used a single genomic profile to represent a cancer cell line while ignoring the rich information contained in the multi-omics data or CCL. The scope of genomic multiomics features is still greatly expandable. Some genomic features that have been proved to be highly informative with cancer have not been integrated and utilized.

To overcome the limitations above, we propose a novel multi-source heterogeneous graph convolutional neural network, referred to as DeepAEG. This architecture encompasses a generic data enhancement module based on sequence recombination and a complete updated graph convolutional neural network with edge features, supplemented by several subnetworks dedicated to advanced feature extraction from multiomics data on drugs and genes, respectively. The combined feature set is input into a one-dimensional convolutional network to complete the regression task of predicting IC50 sensitivity values of CCLs to drugs. The main contributions of this work are summarized as follows:

- We develop a hybrid graph convolutional network by updating both the chemical atom(node) embeddings and bond(edge) embeddings simultaneously. The co-updating strategy provides a new perspective to learn a comprehensive drug representation, ensures that deep information about the drug is completely retained.
- We extend the existing data modality by introducing classical transformer module at the feature extraction level. Transformer module is exploited for capturing the string-based features underlying the SMILES sequences of a drug. The transformer module and GCN module provide complementary information, leading to a more comprehensive drug representation. A more comprehensive characterization and feature extraction of drug molecules has the potential to enhance the accuracy of IC50 predictions and other downstream tasks. Additionally, we extend our multi-omics analysis by introducing copy number variation data, which expands the synergies present in multi-omics profiles of CCLs.
- We propose a molecule augmentation strategy to overcome the vulnerability in the string-based representation by SMILES and enhance the prediction performance. This method can also be applied to other downstream tasks related to drug feature extraction.

## Materials and methods

**Fig. 1:**
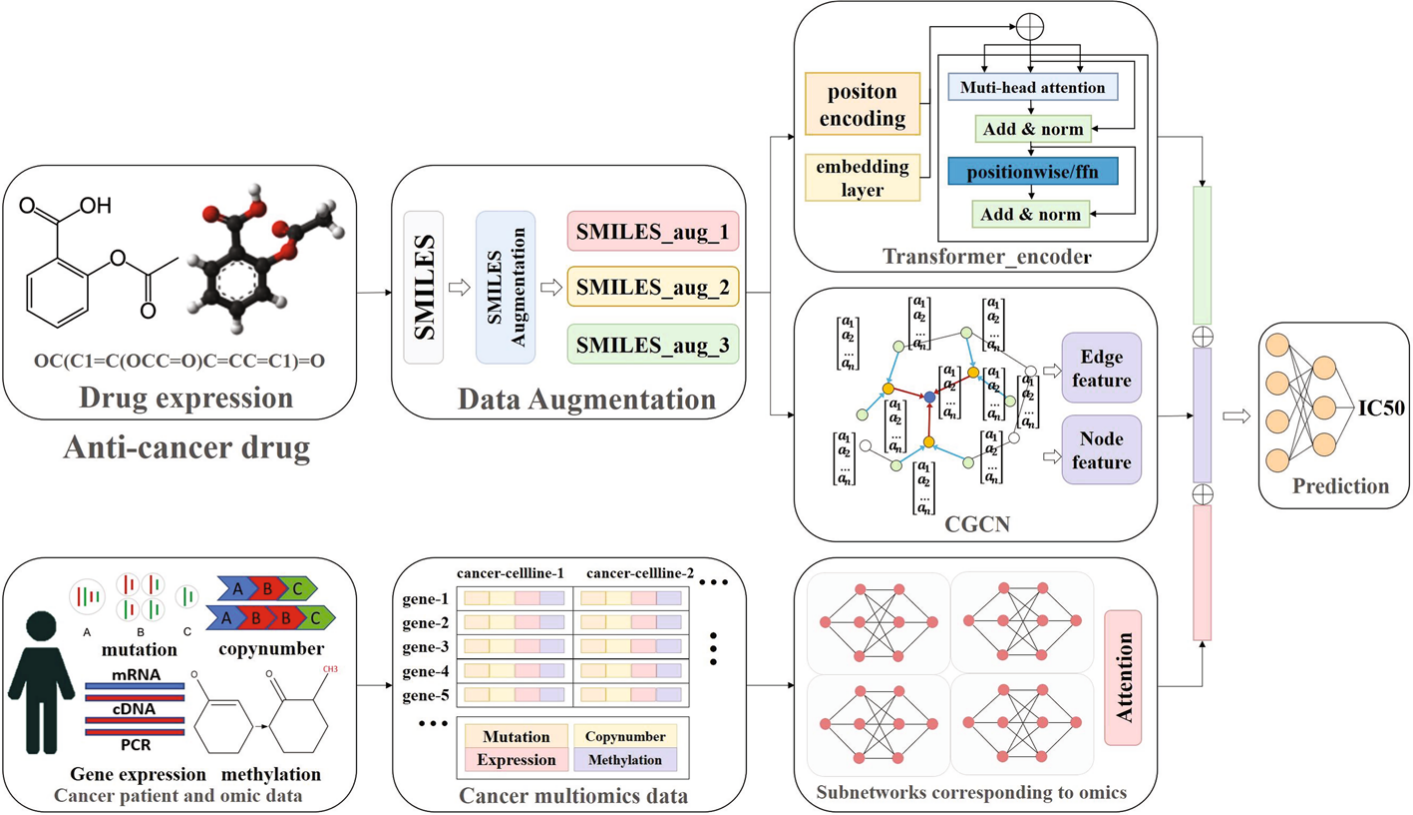
The framework of DeepAEG. DeepAEG mainly includes three parts: data enhancement, drug feature mining and gene expression feature extraction. The drug feature mining module is divided into CGCN module and transformer module. SMILES expression of cancer drugs is data-enhanced after input and converted into advanced latent characterization by the Encoder and Transformer sections of the GCNC, respectively. The features of cancer cell profiles extracted by the sub-networks are connected and input into the classifier network

### Overview of DeepAEG network

We propose an end-to-end deep learning framework including edge update strategy and data enhancement strategy for IC50 prediction, named DeepAEG, which uses transformer and a graph convolutional neural network containing edge information to extract drug characteristics, and combines with four subnetworks to extract advanced information at the cancer omics level (copy number, DNA methylation, gene mutation, Gene expression) to predict the efficacy of anticancer drugs. The structure of the prediction model is shown in Fig. 1.

DeepAEG can incorporate multiple omics features. The model uses a pair of drug-cancer cell line gene profiles and the corresponding ground truth IC50 data and IC50 quantified predicted value as output. On the one hand, the drug is transformed into a higher level of potential expression through graph representation, and on the other hand, vector representation based on substructure sequence extraction can be obtained through transformer. The drug characteristics formed by the two combinations are spliced with the transcriptome information extracted from the four fully connected networks, and then fed into the linear network layer composed of 1D CNN. We use the AdamW optimizer with a learning rate of $1e^{-3}$, batch size of 256, and mean square error as a loss function. The concrete construction of the model are implemented in keras.

### Graph convolutional neural network based drug feature extraction

The unique chemical structure of every drug allows for its natural representation as a graph. Chemical atoms and bonds are represented by the vertices and edges, respectively. So we can naturally represent the drug set as $\left\{ {D}_{n}=\left( {\textbf {V}}_{n}, {\mathcal {A}}_{n}\right) \right| _{n=1}^{M}\}$ where $\textbf{V}_{n}\in {\mathbb {R}}^{N_{n}\times C_{node}}$ and $\textbf{A}_{n}\in {\mathbb {R}}^{N_{n}\times N_{n}\times C_{edge}}$ are the feature matrix and adjacency matrix containing edge information of the *n*th drug. $N_{n}$ is the number of atoms in the *n*th drug, $C_{node}$ and $C_{edge}$ are the number of feature channels of nodes and edges respectively. Each row of the characteristic matrix corresponds to the attribute of an atom. If one element of the adjacent matrix is a one-hot vector, it means that there is an edge between the nodes represented by the horizontal and vertical coordinates and the element is the attribute of the edge. If it is 0, there is no edge. To compensate for the lack of edge information in the UGCN, we introduce a novel CGCN architecture that simultaneously processes node and edge information to comprehensively preserve the deep information in the drug graph representation. The CGCN applied to the *i*th drug is defined as $f({{D}_{i}})\,$ with a layer-wise operation as:

1 $$\begin{aligned} \begin{aligned} V^{(l+1)}=Update\Bigl (V^{l},\sigma (E_{k}^{(l)}V^{l}W_{k}^{(l+1)} +b_{k}^{(l+1)}\\ |k\in (1,\dots ,C_{edge})\Bigl ). \end{aligned} \end{aligned}$$

where $V^{l}$ is the node feature matrix in the *l*th layer, $\sigma (.)$ is the activation function. $W^{l+1}$ and $b^{l+1}$ are learnable parameters. $E_{k}^{l}$ is the *k*th feature matrix of edges. In this representation, the adjacency matrix $A_{n}$ can be viewed as a combination of subadjacency matrices, the number of which is determined by the dimension of the edge features. The adjacency matrix $A_{n}$ can be expressed as:

2 $$\begin{aligned} A_{n} =\left\{ {E}_{n,k}\right| _{k=1}^{c_{edge}}\},{E}_{n,k}\in {\mathbb {R}}^{N_{n}\times N_{n}}. \end{aligned}$$

Furthermore, we update the edge features in two steps. In the first step we define a vector $e_{i,j}$ to represent the relationship between nodes *i* and *j*:

3 $$\begin{aligned} e_{i j}^{(l+1)}=\sigma \Bigl ((V_{i}^{(l+1)}\vert \vert V_{j}^{(l+1)})W_{i j}^{(l+1)}+b_{i j}^{(l+1)}\Bigr ). \end{aligned}$$

$e_{i,j}^{(l+1)}$ is the relation vector between node *i* and node *j* of the *l+1*th layer, $\sigma (.)$ is the activation function,$V_{i}(l)$ and $V_{i}(l+1)$ are the node feature vectors of layer *l* and layer *l+1* respectively, $W^{l+1}_{ij}$ and $b^{l+1}_{ij}$ are learnable parameters. Then, the feature vector $E_{ij}$ of the edge between the node *i* and the node *j* is updated by:

4 $$\begin{aligned} E_{i j}^{(l+1)}=U p d a t e\Big (E_{i j}^{l},e_{i j}^{(l+1)}\Big ). \end{aligned}$$

*Update* represents the update function, $E_{ij}^{l}$ represents the edge feature matrix between node *i* and node *j* in layer *l*. Overall, we extend the work of UGCN by setting *l*=2 as the default to form a two-layer CGCN network. To ensure the effectiveness of the model, only the first layer will be set to carry out edge update steps, while the second layer will not carry out edge update steps. All of the initialization strategies we cover in the discussion section will follow the middle layer with edge updates and the bottom layer without edge updates.

### Transformer based drug feature extraction

In order to avoid the loss of drug molecular information caused by using only molecular descriptors, we introduced Explainable Substructure Partition Fingerprint (ESPFs) to decompose drugs into discrete substructures [25]. The similarity principle has shown that molecules that cause similarity in gene expression in cell lines have equivalent molecular structures or partially overlapping pharmacophores. Therefore we utilize a transformer model to extract substructure data from the input SMILES sequences of drugs.

We first constructed a word collection D containing SMILES string characters and tagged the entire pharmaceutical corpus. We define the tokenized set as T. The labeled set T is updated with the new label at the highest frequency of continuous occurrence until no frequent label exceeds the threshold $\mu$ or the size of D reaches the maximum length $\delta$. This process result in a sequence of substructures $S = \{S_1, S_2,\ldots , S_i\}$ of a drug with *i* atoms. In order to further capture contextual semantic information using encoder module in transformer model, we define substructure sequences as matrices $M^S \in {\mathcal {R}}^{l \times \zeta }$, where *l* represents the length of the substructure, and $\zeta$ represents the maximum length of the substructure sequence of the drug. The *i*th column of the matrix $M_i^{\textrm{S}}$ is a one-hot vector representing the substructure index of the *i*th substructure of the drug sequence. Meanwhile, in order to capture the position information of the drug substructure, we define a one-hot vector $I_i \in R^\zeta$, which representing the location information of the substructure with elements 1 and 0. Therefore, we generate a new representation $D_i$ by summing the representation of the substructure information and the position information of the drug:

5 $$\begin{aligned} {D}_{\textrm{i}}={\textrm{W}}_{c}M_i^{\textrm{s}}+{\textrm{W}}_{p}I_i. \end{aligned}$$

where $W_c$ and $W_p$ are learnable parameters. The potential relationship $Z_{i}$ between the substructures is calculated by the multiple attention layers of the transformer:

6 $$\begin{aligned} Z_{i}=Softmax(\frac{(D_{i}W_{q})(D_{i}W_{k})^{T}}{\sqrt{d}})(D_{i}W_{v}). \end{aligned}$$

The output is fed into a fully connected Feed-forward Network (FFN) to obtain the final expression of each drug:

7 $$\begin{aligned} FFN(x)=max(0,Z_{i}W_{1}+b_{1})W_{2}+b_{2}. \end{aligned}$$

where $W_{1}$, $W_{2}$, $b_{1}$ and $b_{2}$ are learnable parameters.

### Data preparation and data augmentation

The data for our study are aggregated from three publicly available data sets. The GDSC database (www.cancer Rxgene.org) is a comprehensive drug sensitivity database containing drug sensitivity data from multiple cancer types (IC50). The CCLE database contains a large amount of human CCL omics data. The gene expression, gene methylation, gene mutation and copy number data used in the experiment are obtained from this dataset. After excluding drug samples that could not extract SMILES node and edge features, PubChemID and CCLE database data could not be paired, and samples lacking corresponding CCL omics data, a total of 106,496 instances containing 561 cell lines and 221 drugs were finally collected. Considering all the $561\times 221=123981$ drug and cell line interaction pairs, approximately 14.10%(17485) of the IC50 values are missing. Each instance is an interaction between a drug and a CCL and corresponds to a cancer type defined in the Cancer Genome Atlas (TCGA) study. The IC50 values processed through natural log transformed.

SMILES is a molecular encoding method that combines the connectivity structure of a molecule, the atoms and bonds of a molecule, the ring size of a molecule, the stereochemical information of a molecule and other information. Different SMILES expressions may represent the identical molecule, yet exhibit consistent connectivity and molecular structure. This fact makes data augmentation possible in molecular property prediction, which enables the model to better mine the deep information of SMILES and extract task-relevant molecular features. We amplify the SMILES in the task by SMILES permutation [26]. Each augmentation data molecule recombines with the genomic data corresponding to the original molecule to form a new drug-genome pair and participates in the model training. We regard the new data pair as an independent instance to participate in the training. We only performed data augmentation on drug molecules in the training set to avoid data leakage problems in data augmentation.

For the multiomics analysis of CCLs, we consider four main messages from the COSMIC Cancer Gene Screening [27]. For the genome mutation data, binary feature vectors “1” and “0” are used to represent the mutated position and non-mutated position of the gene at the site. The mutation level of the gene is represented by the total number of mutations at multiple sites of the gene. Gene expression data are obtained by log2 conversion of TPM value of gene expression and quantile normalization. DNA methylation data are obtained directly from 1kb bisulfite sequencing data of the upstream TSS promoter. The copy number can be determined by comparing the sample DNA sequence with the reference genome sequence in gene sequencing. The four types of data are processed into a feature matrix after the null data is removed by mean interpolation. Specifically, for CCL $C_{1}$, its expression sequence $X_{c_{1}}$ can be expressed as:

8 $$\begin{aligned} & X_{c1}=\left\{ X_{c1,g1},X_{c1,g2},\ldots ,X_{c1,gn}\right\} \in {\mathbb {R}}^{n\times 4}. \end{aligned}$$

9 $$\begin{aligned}&X_{c1,gi}=\left[ exp_{c1,g{\textbf{i}}},mut_{c1,g{\textbf{i}}},meth_{c1,g{\textbf{i}}}, copy_{c1,g{\textbf{i}}}\right] \in {\mathbb {R}}^{4}, \\&\quad i\in (1,\ldots ,n). \end{aligned}$$

where *n* represents the number of pathogenic genes considered in CCLs, and $X_{c1,gi}$ represents the expression data of the *i*th pathogenic gene in various genomic characteristics of CCLs.

### Gene multigroup subnetwork

On the basis of the original study, we followed the previous practice of Chang et al. [16], combining multiple omics information through a genome-specific neural networks, and we connected the representation of specific omics features learned at the subnetwork layer together through a late integration approach. Each single genomic data is a single element feature matrix composed of genes and Celllines. Since loci are always distributed linearly along chromosomes, and the individual omics data processed have similar data formats, we designed a genomic network consisting of four one-dimensional convolutional networks, each for processing a single genomic data and learning its high-level spatial potential representation. In short, we directly use a fully connected network for feature representation, which is denoted as $\{y_{exp}=f_{exp}(x_{exp}),y_{met}=f_{met}(x_{met}),y_{mut}=f_{mut}(x_{mut}),y_{copy}=f_{copy}(x_{copy})\}$. The expression is used to process the data of gene expression, methylation, gene mutation, and copy number of each sample respectively.

The result can learn the contribution weights of each genome through the attention mechanism. The obtained genomic feature vectors are connected with the drug and cellline learning results to form the final interaction instance feature vectors.

10 $$\begin{aligned} & {\left\{ \begin{array}{ll} f_{exp}:x_{exp}\in {\mathbb {R}}^{1\times d_{exp}}\mapsto y_{exp}\in {\mathbb {R}}^{1\times d}~. \\ f_{met}:x_{met}\in {\mathbb {R}}^{1\times d_{met}}\mapsto y_{met}\in {\mathbb {R}}^{1\times d}~. \\ f_{mut}:x_{mut}\in {\mathbb {R}}^{1\times d_{mut}}\mapsto y_{mut}\in {\mathbb {R}}^{1\times d}~.\\ f_{copy}:x_{copy}\in {\mathbb {R}}^{1\times d_{copy}}\mapsto y_{copy}\in {\mathbb {R}}^{1\times d}~. \end{array}\right. }\end{aligned}$$

11 $$\begin{aligned} & \quad X_{gene}=Attention(y_{exp},y_{met},y_{mut},y_{copy}). \end{aligned}$$

## Results and conclusion

### Performance of DeepAEG

We design a series of experiments to evaluate the performance of our proposed model on cancer drug response prediction. We compared our method with six other methods, including: Ridge Regression, MOLI [19], CDRscan [16], tCNNs [18], DeepCDR [5] and DeepTTA [21]. We create drug and cancer cell profile data from GDSC and CCLE databases and established a set of instances of drug-cancer cell line interactions. These interaction instance sets are shared inputs for all baseline models and DeepAEG. It is important to note that all our baseline methods use the same data set and use the unadjusted hyperparameters of each method as the model Settings to ensure the fairness of model performance comparisons. However, the performance indicators of each baseline model were different from those of the original paper. We employ three commonly used metrics root mean square error (RMSE), Pearson’s correlation coefficient (PCC) and Spearman’s correlation coefficient (SCC) to evaluate the performance of the model. The experiment results are shown in Table 1.

**Table 1:** Performance experiments of IC50 comparison of our method and existing methods

Methods	PCC	SCC	RMSE
Ridge regression	0.780	0.731	2.386
MOLI	0.807 ± 0.007	0.797 ± 0.005	2.081 ± 0.005
CDRscan	0.872 ± 0.004	0.856 ± 0.002	1.941 ± 0.014
tCNNs	0.889 ± 0.015	0.879 ± 0.006	1.781 ± 0.004
DeepCDR	0.9190 ± 0.005	0.8949 ± 0.002	1.082 ± 0.004
DeepTTA	0.9217 ± 0.004	0.8949 ± 0.005	1.0569 ± 0.002
DeepAEG	**0.9333** ± **0.006**	**0.9776** ± **0.005**	**0.9067** ± **0.002**

The best performance values obtained by the model are shown in bold. Three evaluation indexes, including PCC, SCC and RMSE, are selected to evaluate the robustness of the model. DeepAEG consistently achieves optimal performance compared to other past methods

**Fig. 2:**
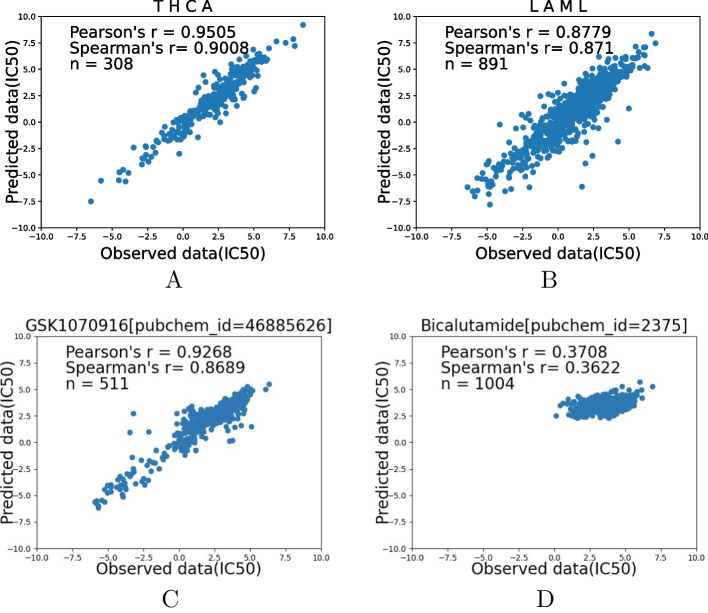
DeepAEG’s performance in different TCGA cancer type predictors and different drugs. The horizontal axis represents the predicted value, and the vertical axis represents the recorded ground truth. DeepAEG showed the best performance in thyroid carcinoma, while the worst-predicted cancer type is Acute Myeloid Leukemia. Also, DeepAEG showed the best performance in GSK1070916, while the worst-predicted drug is the Bicalutamide

### Drug and cell line independent prediction

To further evaluate DeepAEG’s predictive performance, we further applied the model to a single cancer type or a single anticancer drug to evaluate DeepAEG’s performance in specific cancers and specific drugs. To ensure sample richness, 80$\%$ of the examples across 25 cancer types are randomly selected for model training. The remaining 20$\%$ are used for case prediction performance evaluation across multiple TCGA cancers or different drugs. DeepAEG consistently highly performed over all cell lines, with Pearson’s correlation ranging from 0.878 to 0.951. The best predicted case, thyroid carcinoma, and the worst predicted case, Acute Myeloid Leukemia, are shown in Fig. 2A, B.

From the perspective of drugs, we evaluated different classes of drugs to demonstrate the prediction performance of our model for specific drugs. The results indicate that DeepAEG obtains the Pearson’s correlation between all drugs in the range of 0.3708 and 0.9268. The most effective regression predictor is GSK1070916, while the worst predictor is Bicalutamide as shown in Fig. 2C, D.

### Prediction of missing CDRS

**Fig. 3:**
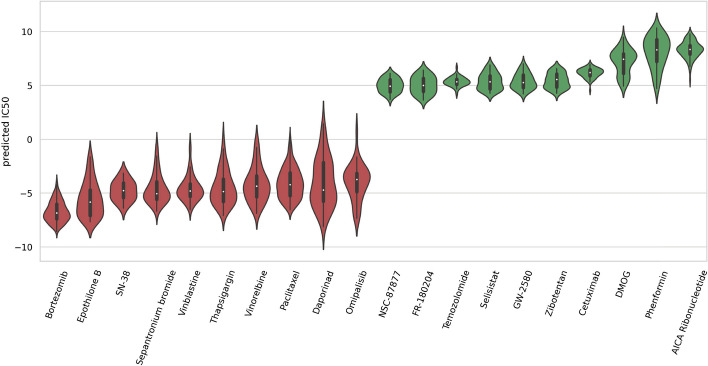
The missing drug-cancer interaction pairs are predicted and ranked according to their average IC50 values, and the ten drugs with the highest and lowest efficacy are selected

Furthermore, we apply DeepAEG to the prediction task of drug-cell line response instances missing from the entire database. To achieve this goal, we trained all 106,494 interactions of all 221 drug classes and 561 cell lines, and predicted 17,485 missing interactions (about 14.1%). We ranked the mean response results of each drug to select the 10 most sensitive and inhibitory drugs. The distribution of IC50 predicted values is shown in Fig. 3. Notably, the predicted optimal drug bortezomib is consistent with the results of the DeepCDR and DeepTTA deletion prediction experiments. Bortezomib, also known as S-341, is the first boron-containing drug that was approved by the FDA in 2003 for the treatment of multiple myeloma [28]. Bortezomib has been shown to be an active proteasome inhibitor in a variety of CCLs. Anti-cancer mechanisms of bortezomib elucidated by preclinical studies include: Up-regulate proapoptotic proteins (e.g., Noxa, I$\varkappa$B), inhibit NF$\varkappa$B and its anti-apoptotic target genes, inhibit multiple anti-apoptotic proteins (e.g., Bcl-XL, Bcl-2 and STAT-3), and down-regulate the expression of several proteins involved in DNA repair pathways, and induced endoplasmic reticulum (ER) stress and pro-apoptotic unfolded protein response (UPR). Bortezomib has a powerful chemical/radiosensitization effect that can overcome traditional tumor resistance when used in combination with potential chemotherapy [29]. Ebomycin is a class of 16-element macrolide compounds that is easy to synthesize. It has been proved that Ebomycin induces apoptosis of human breast cancer cells by down-regulating anti-apoptotic protein [30]. The drug is also highly active against cancer cells that are resistant to paclitaxel and other anti-cancer drugs [31]. We also found Phenformin and AICA ribonucleotide as the two least effective drugs like DeepCDR [5], but both of them have been proved to be effective anticancer drugs [32, 33], and their anticancer effects may be achieved by influencing the cellular environment or combination therapy [34].

**Fig. 4:**
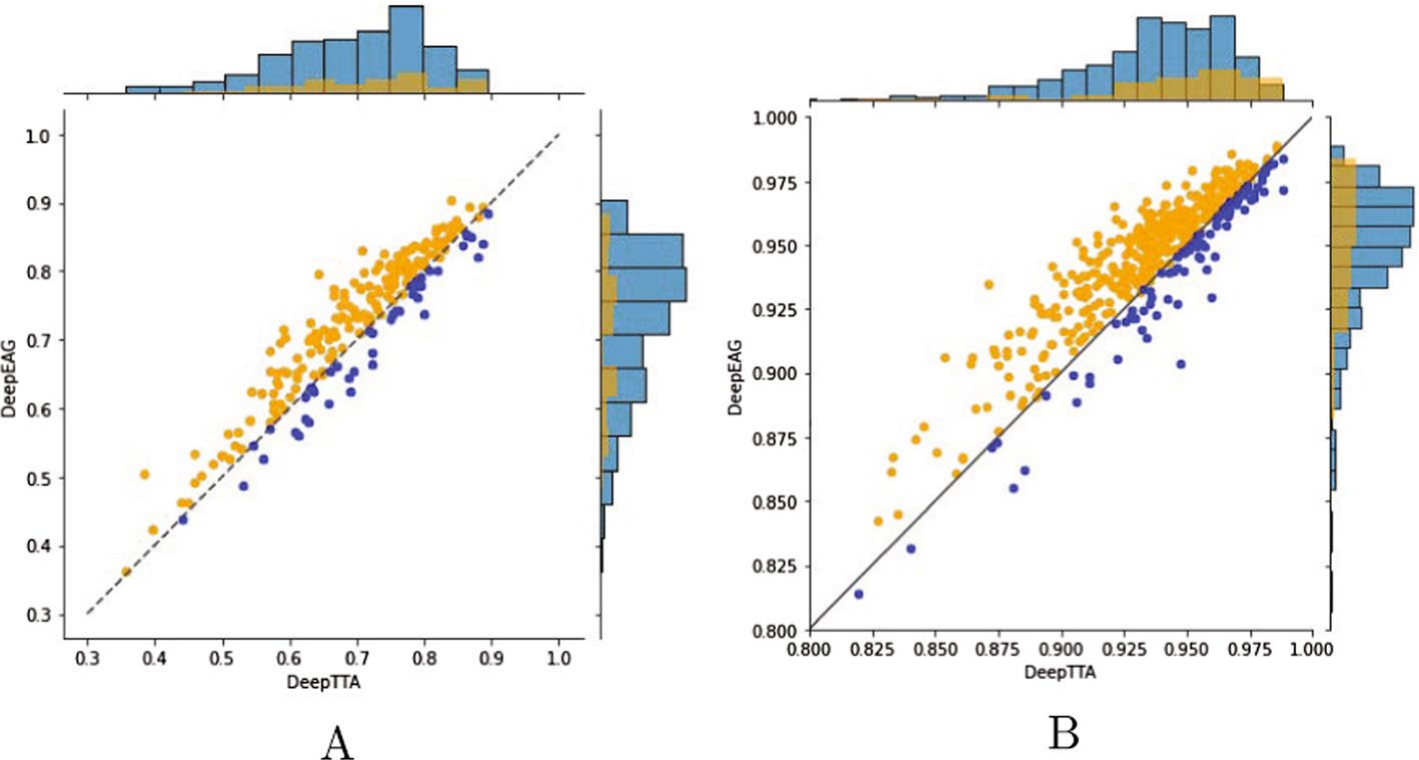
Pearson’s correlation results for DeepAEG and DeepTTA in independence tests. Each scatter represents a drug or cellline, and its horizontal and vertical coordinates represent Pearson’s correlation results for DeepTTA and DeepAEG, respectively. The scatter in the upper left corner of the function $Y=X$ is the case where DeepAEG is better than DeepTTA

### Independent experiments on drugs and cell lines

Further, we apply DeepAEG to cellline and drug independence tests to assess its capabilities. The SOTA model DeepTTA is used as a baseline for comparison. DeepAEG achieved better performance in both cellline and drug independence tests, as shown in Fig. 4A, B. The results of blind drug testing decrease significantly compared with the previous experiment results, but still achieved the average Pearson’s correlation result of 0.726, which is better than that for DeepTTA’s, and 79.2% of the average results of 221 drugs are better than that of DeepTTA’s. For cellline tests, Pearson’s correlation is 0.945 on average, better than that for DeepTTA’s, and 81.2% of 494 celllines are better than DeepTTA’s.

**Table 2:** Model ablation studies with different experiment settings

Trainning settings	PCC
Drug feature only	0.8507
Drug feature w/ muti-omics	0.9255
Drug feature w/ muti-omics w/ T	0.9268
Drug feature w/ muti-omics w/ T w/ Edge	0.9304
Double data enhancement w/ muti-omics w/ T w/ Edge	0.9318
Triple data enhancement w/ muti-omics w/ T w/ Edge	**0.9333**

We show the contribution of the edge update module, transformer module and data enhancement features. Each module provides a PCC improvement of between 0.3 and 0.5% for the final results

Muti-omics represents Multiomics characteristics of genes and T represents the transformer module. Edge represent the edge feature update module

The best performance values obtained by the model are shown in bold

**Table 3:** Top-5 prioritized cancer-associated genes identified by DeepAEG

Drug	Cell line	TCGA type	Top-5 related genes
Erlotinib	A3/KAW	DLBC	MTOR,ALK,RAF1,ETV5,**EGFR**
Lapatinib	HCC1599	BRCA	MDS2,FLCN ,**EGFR** ,VHL,FCGR2B
Crizotinib	GA-10	DLBC	ARHGEF10L,CDX2,CDG10,**MET**,CANT1
Dasatinib	NALM-6	ALL	IKBKB,THRAP3,CDKN1A,BCL2L12,**SRC**
Imatinib	KG-1	LAML	FOXR1,SFPQ,ERCC5,RAD17,**ABL**

We employed neural network automatic differentiation to compute the sum of gradients and rank the absolute value of each gene. Notably, certain top-5 genes have been corroborated by previous studies as being associated with drug responses or diseases corresponding to specific cell lines. These genes are indicated in bold

### Model ablation analysis

We propose multiple methods to capture the underlying information of drug molecules, and it is necessary to evaluate each module’s contribution to the final result. For our model ablation analysis, we investigate the performance of including edge features, transformer module and data augmentation and conduct de novo training of DeepAEG respectively. The results indicate that edge information only contributes slightly to overall Pearson’s correlation. Feature extraction with a single Transformer module yields similar performance compared to GCN without edge features. Combining Transformer and CGCN modules improves model performance, surpassing either module alone. To further improve model performance through data enhancement, we apply Transformer and CGCN modules, doubling and tripling the data enhancement by reassembling each drug molecule SMILES into two or three virtual SMILES sequences, respectively. The specific results are shown in Table 2. Results show that our data enhancement strategy significantly enhances model performance.

### DeepAEG predicts cancer-related genes

In order to assess the performance of DeepAEG in acquiring biological knowledge and investigate its application in the field of biology, we adopted a methodology similar to DeepCDR [5]. Given a specific drug and cell line pair, we ranked the genes based on their contributions to the output results and evaluated the relevance of specific genes. In order to achieve this, we use the idea of multivariate function deflection to calculate the gradient of the input single omics gene spectrum by using the output IC50 as a benchmark, and the calculated single omics gradient vector length is consistent with the number of genes, and finally sorted by the gradient vectors of the four genomic data features. Our entire neural network model can be expressed as:

12 $$\begin{aligned} y = g(f_{\text {exp}}(\text {exp}), f_{\text {met}}(\text {met}), f_{\text {mut}}(\text {mut}), f_{\text {copy}}(\text {copy}), f_{\text {drug}}(\text {drug})) \end{aligned}$$

For the *i*th gene, the sum of absolute values of its gradient can be expressed as:

13 $$\begin{aligned} \text {GradSum}_i = \left| \frac{\partial y}{\partial \text {exp}_i}\right| + \left| \frac{\partial y}{\partial \text {met}_i}\right| + \left| \frac{\partial y}{\partial \text {mut}_i}\right| + \left| \frac{\partial y}{\partial \text {copy}_i}\right| \end{aligned}$$

Each partial derivative can be obtained by automatic differential calculation. And we can find the most influential genes by comparing the GradSum values of all genes:

14 $$\begin{aligned} i_{\text {max}} = \arg \max _i (\text {GradSum}_i) \end{aligned}$$

The five examples of drug-cancer cell line interaction genes we highlighted are shown in Table 3. We find that many of the genes at the top of the list were confirmed to be oncogenes or involved in the mechanism of drug action. The drug Erlotinib has been proved to be effective in the treatment of breast cancer and lymphoma [35, 36]. Erlotinib has been substantiated as a potent inhibitor of EGFR [37], and its gene expression has also been demonstrated to be correlated with several cancers [38, 39]. This finding is also consistent with the results obtained in DeepCDR [5]. Furthermore, Crizotinib, initially developed as a MET inhibitor, has demonstrated efficacy for lymphoma kinase-positive lymphoma patients [40]. Our results on the Crizotinib-GA-10 interaction case showed that MET is the fourth relevant gene and it was shown to be specifically expressed in this cancer [41]. Besides, Dasatinib has become a potential treatment for acute lymphoblastic leukemia by acting on SRC [42], while the Src family of non-receptor tyrosine kinases has been identified as a potential mediator for BCR-ABL-induced leukemia [43, 44]. Imatinib therapy targeting the oncogene product BCR-ABL has transformed chronic myelogenous leukemia (CML) from a life-threatening disease to a chronic condition. Two related genes, SRC and ABL, appeared in the top-5 related genes [45]. In conclusion, our model demonstrates the capability to uncover interrelationships among drug-disease-related gene triads and has the ability to facilitate the discovery of potential therapeutic targets. This ability holds significant potential in advancing the development of precise clinical treatments and targeted cancer medications.

## Conclusion

In this study, we developed an end-to-end deep learning model called DeepAEG to accurately predict anti-cancer drug responses. To the best of our knowledge, DeepAEG is the first method to apply edge information characteristics and data enhancement to cancer drug response problems. Comprehensive experiments showed that fusion of edge information features, SMILES sequence recombination, and expanded multiomics maps optimized the feature extraction capability of drug-cell line reaction instances. DeepAEG shows the best PCC, SCC and RMSE. And the result of the missing data prediction also identified the potential effective drug (bortezomib, AICA) and the most relevant genes. The results highlights DeepAEG’s predictive power and its potential value in guiding cancer-specific therapies. We leave the following work as our future research directions. (1) Since coordinates can quantify bond length between two atoms, and there is a specific power law relationship between bond length, bond strength and electron density distribution, three-dimensional molecular coordinate expression can enrich drug molecular information and potentially improve model prediction performance. (2) Through the knowledge map of cancer cells, the integration and fusion of knowledge in different fields can be realized, so as to meet the requirements for the integration and application of multidisciplinary knowledge in the context of cancer precision medicine. Using DeepAEG as a guide, DeepAEG contributes to the growing field of precision medicine, facilitating cancer mechanism research and specific drug development.

## Data Availability

DeepAEG is freely available at https://github.com/zhejiangzhuque/DeepAEG.

## References

[1] Sung H, Ferlay J, Siegel RL, Laversanne M, Soerjomataram I, Jemal A, Bray F (2021). Global cancer statistics 2020: GLOBOCAN estimates of incidence and mortality worldwide for 36 cancers in 185 countries. CA Cancer J Clin.

[2] Mansoori B, Mohammadi A, Davudian S, Shirjang S, Baradaran B (2017). The different mechanisms of cancer drug resistance: a brief review. Adv Pharm Bull.

[3] Verma M (2012). Personalized medicine and cancer. J Pers Med.

[4] Volm M, Efferth T (2015). Prediction of cancer drug resistance and implications for personalized medicine. Front Oncol.

[5] Liu Q, Hu Z, Jiang R, Zhou M. DeepCDR: a hybrid graph convolutional network for predicting cancer drug response. Bioinformatics. 2020;36(Supplement_2):911–8. 10.1093/bioinformatics/btaa822 33381841

[6] Iorio F, Knijnenburg TA, Vis DJ, Bignell GR, Menden MP, Schubert M, Aben N, Gonçalves E, Barthorpe S, Lightfoot H (2016). A landscape of pharmacogenomic interactions in cancer. Cell.

[7] Gagan J, Van Allen EM (2015). Next-generation sequencing to guide cancer therapy. Genome Med.

[8] Yang W, Soares J, Greninger P, Edelman EJ, Lightfoot H, Forbes S, Bindal N, Beare D, Smith JA, Thompson IR (2012). Genomics of Drug Sensitivity in Cancer (GDSC): a resource for therapeutic biomarker discovery in cancer cells. Nucleic Acids Res.

[9] Barretina J, Caponigro G, Stransky N, Venkatesan K, Margolin AA, Kim S, Wilson CJ, Lehár J, Kryukov GV, Sonkin D (2012). The Cancer Cell Line Encyclopedia enables predictive modelling of anticancer drug sensitivity. Nature.

[10] Celebi R, Bear Do’t n Walk IV O, Movva R, Alpsoy S (2019). In-silico prediction of synergistic anti-cancer drug combinations using multi-omics data. Sci Rep.

[11] Romero Hung J, Li C, Wang T, Guo J, Wang P, Shao C, Wang J, Shi G, Liu X, Wu H (2023). Dragon: dynamic recurrent accelerator for graph online convolution. ACM Trans Des Autom Electron Syst.

[12] Zhang N, Wang H, Fang Y, Wang J, Zheng X, Liu XS (2015). Predicting anticancer drug responses using a dual-layer integrated cell line-drug network model. PLoS Comput Biol.

[13] Peng W, Chen T, Dai W (2021). Predicting drug response based on multi-omics fusion and graph convolution. IEEE J Biomed Health Inform.

[14] Geeleher P, Cox NJ, Huang RS (2014). Clinical drug response can be predicted using baseline gene expression levels and in vitro drug sensitivity in cell lines. Genome Biol.

[15] Dong Z, Zhang N, Li C, Wang H, Fang Y, Wang J, Zheng X (2015). Anticancer drug sensitivity prediction in cell lines from baseline gene expression through recursive feature selection. BMC Cancer.

[16] Chang Y, Park H, Yang H-J, Lee S, Lee K-Y, Kim TS, Jung J, Shin J-M (2018). Cancer drug response profile scan (CDRscan): a deep learning model that predicts drug effectiveness from cancer genomic signature. Sci Rep.

[17] Daemen A, Griffith OL, Heiser LM, Wang NJ, Enache OM, Sanborn Z, Pepin F, Durinck S, Korkola JE, Griffith M (2013). Modeling precision treatment of breast cancer. Genome Biol.

[18] Liu P, Li H, Li S, Leung K-S (2019). Improving prediction of phenotypic drug response on cancer cell lines using deep convolutional network. BMC Bioinform.

[19] Sharifi-Noghabi H, Zolotareva O, Collins CC, Ester M (2019). MOLI: multi-omics late integration with deep neural networks for drug response prediction. Bioinformatics.

[20] Ma T, Liu Q, Li H, Zhou M, Jiang R, Zhang X (2022). DualGCN: a dual graph convolutional network model to predict cancer drug response. BMC Bioinform.

[21] Jiang L, Jiang C, Yu X, Fu R, Jin S, Liu X (2022). DeepTTA: a transformer-based model for predicting cancer drug response. Brief Bioinform.

[22] Krenn M, Häse F, Nigam A, Friederich P, Aspuru-Guzik A (2020). Self-referencing embedded strings (SELFIES): a 100% robust molecular string representation. Mach Learn: Sci Technol.

[23] Wang H, Dai C, Wen Y, Wang X, Liu W, He S, Bo X, Peng S (2023). GADRP: graph convolutional networks and autoencoders for cancer drug response prediction. Brief Bioinform.

[24] Weininger D (1988). Smiles, a chemical language and information system. 1. Introduction to methodology and encoding rules. J Chem Inf Comput Sci.

[25] Huang K, Xiao C, Glass L, Sun J. Explainable substructure partition fingerprint for protein, drug, and more. In: NeurIPS learning meaningful representation of life workshop. 2019.

[26] Wu Z, Jiang D, Wang J, Zhang X, Du H, Pan L, Hsieh C-Y, Cao D, Hou T (2022). Knowledge-based BERT: a method to extract molecular features like computational chemists. Brief Bioinform.

[27] Sondka Z, Bamford S, Cole CG, Ward SA, Dunham I, Forbes SA (2018). The COSMIC Cancer Gene Census: describing genetic dysfunction across all human cancers. Nat Rev Cancer.

[28] Richardson PG, Barlogie B, Berenson J, Singhal S, Jagannath S, Irwin D, Rajkumar SV, Srkalovic G, Alsina M, Alexanian R (2003). A phase 2 study of bortezomib in relapsed, refractory myeloma. N Engl J Med.

[29] Mujtaba T, Dou QP (2011). Advances in the understanding of mechanisms and therapeutic use of bortezomib. Discov Med.

[30] Wittmann S, Bali P, Donapaty S, Nimmanapalli R, Guo F, Yamaguchi H, Huang M, Jove R, Wang HG, Bhalla K (2003). Flavopiridol down-regulates antiapoptotic proteins and sensitizes human breast cancer cells to epothilone B-induced apoptosis. Can Res.

[31] Cheng H, Huang H, Huang G (2018). Synthesis and antitumor activity of epothilone B. Eur J Med Chem.

[32] Miskimins WK, Ahn HJ, Kim JY, Ryu S, Jung Y-S, Choi JY (2014). Synergistic anti-cancer effect of phenformin and oxamate. PLoS ONE.

[33] Su C-C, Hsieh K-L, Liu P-L, Yeh H-C, Huang S-P, Fang S-H, Cheng W-C, Huang K-H, Chiu F-Y, Lin I-L (2019). AICAR induces apoptosis and inhibits migration and invasion in prostate cancer cells through an AMPK/mTOR-dependent pathway. Int J Mol Sci.

[34] Jafari-Gharabaghlou D, Pilehvar-Soltanahmadi Y, Dadashpour M, Mota A, Vafajouy-Jamshidi S, Faramarzi L, Rasouli S, Zarghami N (2018). Combination of metformin and phenformin synergistically inhibits proliferation and hTERT expression in human breast cancer cells. Iran J Basic Med Sci.

[35] Murray Stewart T, Von Hoff D, Fitzgerald M, Marton LJ, Becerra CHR, Boyd TE, Conkling PR, Garbo LE, Jotte RM, Richards DA (2021). A Phase Ib multicenter, dose-escalation study of the polyamine analogue PG-11047 in combination with gemcitabine, docetaxel, bevacizumab, erlotinib, cisplatin, 5-fluorouracil, or sunitinib in patients with advanced solid tumors or lymphoma. Cancer Chemother Pharmacol.

[36] Sharma P, Khan Q, Kimler B, Klemp J, Connor C, McGinness M, Mammen J, Tawfik O, Fan F, Fabian C. Abstract P1-11-07: results of a Phase II study of neoadjuvant platinum/taxane based chemotherapy and erlotinib for triple negative breast cancer. Cancer Res. 2010;70(24_Supplement):1–11.

[37] Sayar BS, Rüegg S, Schmidt E, Sibilia M, Siffert M, Suter MM, Galichet A, Müller EJ (2014). EGFR inhibitors erlotinib and lapatinib ameliorate epidermal blistering in pemphigus vulgaris in a non-linear, V-shaped relationship. Exp Dermatol.

[38] Li Y, Jia Z, Zhao H, Liu X, Luo J, Cui G, Kong X (2021). TUC338 promotes diffuse large B cell lymphoma growth via regulating EGFR/PI3K/AKT signaling pathway. J Oncol.

[39] Leek RD, Hunt NC, Landers RJ, Lewis CE, Royds JA, Harris AL (2000). Macrophage infiltration is associated with VEGF and EGFR expression in breast cancer. J Pathol: J Pathol Soc G B Irel.

[40] Gambacorti Passerini C, Farina F, Stasia A, Redaelli S, Ceccon M, Mologni L, Messa C, Guerra L, Giudici G, Sala E (2014). Crizotinib in advanced, chemoresistant anaplastic lymphoma kinase-positive lymphoma patients. J Natl Cancer Inst.

[41] Lam BQ, Dai L, Qin Z (2016). The role of HGF/c-MET signaling pathway in lymphoma. J Hematol Oncol.

[42] Konig H, Copland M, Chu S, Jove R, Holyoake TL, Bhatia R (2008). Effects of dasatinib on SRC kinase activity and downstream intracellular signaling in primitive chronic myelogenous leukemia hematopoietic cells. Can Res.

[43] Klejman A, Schreiner SJ, Nieborowska-Skorska M, Slupianek A, Wilson M, Smithgall TE, Skorski T (2002). The Src family kinase Hck couples BCR/ABL to STAT5 activation in myeloid leukemia cells. EMBO J.

[44] Danhauser-Riedl S, Warmuth M, Druker BJ, Emmerich B, Hallek M (1996). Activation of Src kinases p53/56 lyn and p59 hck by p210 BCR/ABL in myeloid cells. Can Res.

[45] Corbin AS, Agarwal A, Loriaux M, Cortes J, Deininger MW, Druker BJ (2011). Human chronic myeloid leukemia stem cells are insensitive to imatinib despite inhibition of BCR-ABL activity. J Clin Investig.

